# Pre- and post-treatment of α-Tocopherol on cognitive, synaptic plasticity, and mitochondrial disorders of the hippocampus in icv-streptozotocin-induced sporadic Alzheimer’s-like disease in male Wistar rat

**DOI:** 10.3389/fnins.2023.1073369

**Published:** 2023-04-20

**Authors:** Fatemeh Nabavi Zadeh, Maryam Nazari, Abdollah Amini, Soheila Adeli, Amir Barzegar Behrooz, Javad Fahanik Babaei

**Affiliations:** ^1^Electrophysiology Research Center, Neuroscience Institute, Tehran University of Medical Sciences, Tehran, Iran; ^2^Department of Physiology, School of Medicine, Tehran University of Medical Sciences, Tehran, Iran; ^3^Neurophysiology Research Center, Shahid Beheshti University of Medical Sciences, Tehran, Iran; ^4^Department of Biology and Anatomical Sciences, School of Medicine, Shahid Beheshti University of Medical Sciences, Tehran, Iran

**Keywords:** Alzheimer’s disease, α-Tocopherol, mitochondrial dysfunction, synaptic plasticity, stress oxidative

## Abstract

**Objective:**

Most dementia cases in the elderly are caused by Alzheimer’s disease (AD), a complex, progressive neurological disease. Intracerebroventricular (ICV) administration of streptozotocin (STZ) in rat’s results in aberrant brain insulin signaling, oxidative stress, and mitochondrial dysfunction that impair cognition change neural plasticity, and eventually lead to neuronal death. The current study aims to define the neuroprotective action of alpha-tocopherol in enhancing mitochondrial function and the function of synapses in memory-impaired rats brought on by icv-STZ.

**Methods:**

Male Wistar rats were pre-treated with (α-Tocopherol 150 mg/kg) orally once daily for 7 days before and 14 days after being bilaterally injected with icv-STZ (3 mg/kg), while sham group rats received the same volume of STZ solvent. After 2 weeks of icv-STZ infusion, rats were tested for cognitive performance using a behaviors test and then were prepared electrophysiology recordings or sacrificed for biochemical and histopathological assays.

**Results:**

The cognitive impairment was significantly minimized in the behavioral paradigms for those who had taken α-Tocopherol. In the hippocampus of icv-STZ rat brains, α-Tocopherol ocopherol effectively prevented the loss of glutathione levels and superoxide dismutase enzyme activity, lowered mitochondrial ROS and mitochondrial membrane potential, and also brought about a decrease in Aβ aggregation and neuronal death.

**Conclusion:**

Our findings demonstrated that by lowering neurobehavioral impairments caused by icv-STZ, oxidative stress, and mitochondrial dysfunction, α-Tocopherol enhanced intracellular calcium homeostasis and corrected neurodegenerative defects in the brain. These findings examine the available approach for delaying AD connected to mitochondrial malfunction and plasticity issues.

## Graphical Abstract

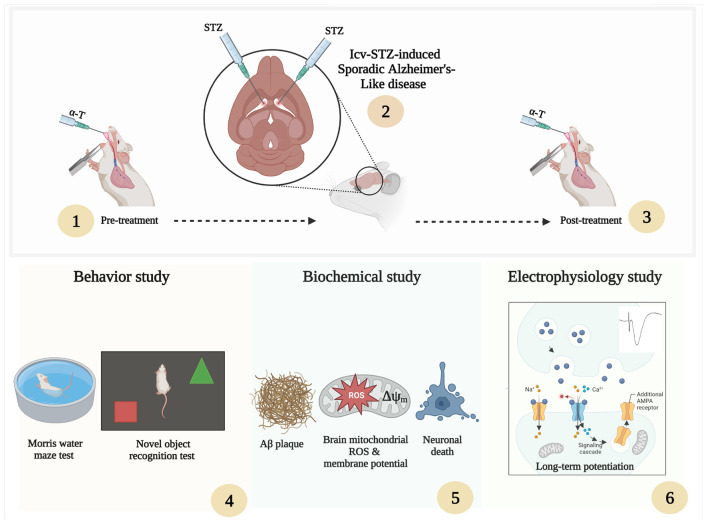

## 1. Introduction

Most dementia in the elderly is caused by AD, a multifactorial, age-related, progressive neurodegenerative disorder (Anand et al., 2014). It is commonly known that among the familial (fAD) and sporadic (sAD) types of this illness, sporadic Alzheimer’s disease affects more than 95% of those involved. Although its origin is uncertain, environmental, genetic, and metabolic, risk factors might be interesting (Reitz et al., 2011; Chakrabarti et al., 2015). In addition to the amyloid cascade hypothesis, which proposes the toxic actions of misfolded and oligomerized amyloid beta peptide (Aβ), there are several typical molecular hallmarks of AD pathogenesis, including altered tau phosphorylation, oxidative damage, mitochondrial dysfunction, neuroinflammation, and decreased glucose utilization. The loss of brain connections, apoptosis, and gradually declining behavioral and cognitive skills were the eventual effects of these molecular markers (Kraska et al., 2012; Iqbal et al., 2014; Sorrentino et al., 2014; Nazem et al., 2015; Grieb, 2016).

Initially thought to be an antibiotic, STZ is harmful to pancreatic beta cells and is frequently used to cause experimental diabetes. Numerous investigations have demonstrated that administering a modest dosage of STZ through an icv injection causes disturbed brain insulin signaling balance and a deficiency in cerebral glucose metabolism. In addition, icv injection of STZ increases cerebral aggregated amyloid beta peptide 1–42 (Aβ_1–42_) and total tau protein slowly and over a long period. Eventually, it can lead to memory impairment, biochemical alterations, mitochondria disorder, neural plasticity impairment, and neuroinflammation. STZ is a valid experimental model of early pathophysiological changes in AD based on evidences (Salkovic-Petrisic and Hoyer, 2007; Labak et al., 2010; Santos et al., 2012; Chen et al., 2013; Correia et al., 2013; Grieb, 2016; Kamat et al., 2016; Park et al., 2020).

Plasticity impairment in pathological conditions is one of the relevant targets of neuroscience. The hippocampus is a vulnerable structure that can be impaired by endogenous and exogenous factors and events, including stroke, head trauma, and AD (McEwen, 1999). Long-term potentiation (LTP) is the hippocampus’s ability to exhibit functional synaptic plasticity (Bliss and Lomo, 1973), and it is one of the main neural mechanisms by which memory is stored in the brain (Maren and Baudry, 1995; Lynch, 2004). Studies have shown that nutrients, such as vitamin E [α-Tocopherol (α-T)], may control mental functions, affect neuroplasticity, and affect several elements of plasticity-related processes in the healthy and pathological brain (Ambrogini et al., 2016). Long-term hippocampal potential (LTP), a cellular substrate for mammalian learning and memory, has been shown in animal models to be impaired in conditions associated with increased levels of oxidative stress, mitochondrial dysfunction, and apoptosis. However, vitamin E can buffer or prevent this decline (Bliss and Collingridge, 1993; Malenka and Nicoll, 1999). Previous studies reveal that increased ROS generation and increase in mitochondrial membrane permeability due to mitochondrial ROS affect neurons and are strongly associated with apoptosis markers and cell death–mediated the pathogenesis of neurodegenerative disorders, such as AD (Morimoto, 2008; Wang et al., 2014). For this reason, attention has been paid to a wide variety of natural antioxidants, such as vitamins, and their production sources to scavenge free radicals and protect neurons from oxidative damage (Mansouri et al., 2013; Fahanik-Babaei et al., 2019).

Vitamin E (as a powerful antioxidant) was proposed as a treatment for AD many years ago; however, its effectiveness is still not apparent. α-Tocopherol is a dominant form of vitamin E found in tissues and is present in many fruits, vegetables, and plants (McLaughlin and Weihrauch, 1979; Jiang, 2014). α-Tocopherol acts as a radical-scavenging antioxidant to protect cells against ROS (Ulatowski and Manor, 2015). However, the biological activities of α-Tocopherol are not limited to its antioxidant actions. It also has hypocholesterolemia properties, anti-inflammatory, anticancer, antiangiogenic, and neuroprotective roles in various neurodegenerative disorders (Kumar et al., 2019; Lloret et al., 2019), driving its importance for brain health.

Although, in the last few decades, several animal and clinical studies have been conducted on the effects of α-Tocopherol and have appeared to have abundant benefits in ameliorating neurodegenerative diseases, especially AD, however, α-Tocopherol effects on improved function mitochondria and the involvement of synaptic function and neuronal plasticity remain unexplored in STZ induced memory-impaired rats. On the other hand, there is a requirement for additional information on the potential relationships between α-Tocopherol and other factors that may influence cognition. Therefore, in the present study, we examined the protective effects of α-Tocopherol on pre- and post-treatment cognitive, synaptic plasticity, and mitochondrial disorders of the hippocampus in icv-streptozotocin induced sAD in male Wistar rats. In this study, we also compare the potential effects of α-Tocopherol in reducing the level of Aβ plaques and cell death and their modulation on behavioral functions in the sAD model. This study may indicate the potential application of α-Tocopherol in developing therapeutic strategies for AD by targeting the mitochondria dysfunction pathway, and synaptic plasticity alters.

## 2. Materials and methods

### 2.1. Animal

One hundred two male adult albino Wistar rats at the age of 10–12 weeks and a weight range of 220–250 g were housed in Plexiglas cages with woodchip bedding in groups of 3–4 per cage at a room temperature of 22 ± 1°C under a standard 12–12 h light/dark cycle. Animals were allowed to acclimate to their environment for 7 days before being tested and handled daily. All experiments were carried out between 9 a.m. till 3 p.m. Food and water were available *ad libitum*. Procedures involving animals and their care were conducted in conformity with the National Institutes of Health guidelines for the care and use of laboratory animals (NIH No: 8023, revised 1978). In addition, this experiment was approved by the Ethics and Research Committee of the Tehran University of Medical Sciences (IR TUMS.NI.REC.1398.056).

### 2.2. Surgical induction of sporadic Alzheimer’s disease using ICV-STZ injection

**FIGURE 1:**
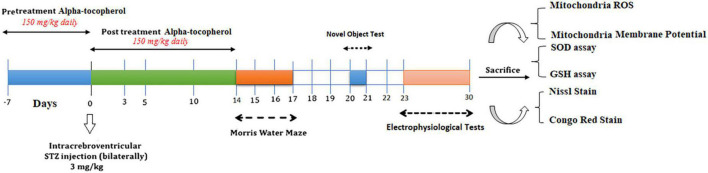
Parameters measured and the time intervals are presented in a scheme of experimental diagram.

The rats were randomly allocated to the following six equal-sized groups (*n* = 17 per group): Sham, Sham + α-Tocopherol 7 day (pre-treatment) and 14 days (post-treatment) (150 mg/kg daily), STZ, STZ + solvent (sesame oil), STZ + α-Tocopherol 7 (pre-treatment) and STZ + α-Tocopherol 14 (post-treatment). STZ was dissolved in distilled water and administered at a single dose of 3 mg/kg. The STZ dosage was chosen prior to research using the rat model of sAD (Tiwari et al., 2009; Correia et al., 2013; Rai et al., 2014). Sham animals received an equivalent volume of the vehicles (normal saline). After using a mixture of ketamine (100 mg/kg) and xylazine (5 mg/kg) to anesthetize the animals, the rats were mounted in a stereotaxic frame. A single bilateral icv injection of streptozotocin (STZ, 3 mg/kg bilaterally 2.5 μl each ventricle) was done for 5 min on each side (0.8 mm posterior to the bregma, ±1.5 mm lateral to the sagittal suture, and 3.6 mm below the dura (Paxinos and Watson, 2006). After injection, the needle was left in place for an additional 60 s. Then the skin was sutured, and the animals were monitored before returning to their home cages. The groups receiving α-Tocopherol started receiving it 24 h after STZ injection. Behavior tests were conducted on week two after STZ microinjection on experimental rat groups. Figure 1 shows a schematic diagram of the experimental design of the study.

### 2.3. Neurobehavioral evaluation

#### 2.3.1. Morris water maze

This study used the Morris water maze (MWM) to examine rats’ cognitive function, especially spatial learning and memory. All animals were looked at after a 2-week STZ injection and were implemented as previously described (Morris et al., 1990). Briefly, the maze consisted of a round pool (110 cm in radius and 70 cm in height) filled with tap water (22 ± 1°C). The maze was divided into four equal quadrants, and a hidden circular platform was located at the center of one of the quadrants. In the present study, the MWM was conducted over four consecutive days. The task consisted of two stages: the acquisition phase for spatial learning assessment (four trials daily) and the probe test for retention retrieval of memory (one trial). In the acquisition phase, the animal was dropped into the water in the different quadrants and given 90 s to seek the hidden platform in each trial. Otherwise, it was guided to reach the platform and stayed that for 30 s; escape latency was recorded at 90 s. A 60-s probe test with the hidden platform removed was also given 24 h after the completion of the last session. The time that the rat spent on the platform (escape latency) and the movement to find the hidden platform (Distance traveled) were accounted for to assess the ability of spatial learning. Time spent in the target quadrant and the latency of the first entry to the target quadrant were the parameters analyzed for the probe test. The path the animal swam was automatically recorded using a video camera-based system (EthoVision, Noldus, version 14).

#### 2.3.2. Novel object recognition test

The novel object recognition test (NORT) was applied to examine cognitive function in rats. The test included 3 days and three phases: the habituation phase, the familiarization phase, and the test phase. In the first phase, a rat had not displayed a preference for any objects in 5 min. In the second phase, the rats were exposed to the same two objects for 5 min. In the third phase, one of the objects was replaced by a novel object. After each trial, the objects and maze were carefully cleaned with ethanol (10%). The total exploring time for each object was automatically recorded using a video camera-based System (EthoVision, Noldus, Version 14). Exploratory behaviors were performed as sniffing, licking, and climbing within a range of 2 cm or less from the object. The total time exploring two novel and familiar objects and Recognition Index (RI) was measured by comparing the time spent investigating the novel object relative to the total object investigation [RI = TN/(TN + TF)] (RI) (Antunes and Biala, 2012).

### 2.4. Biochemical estimations

#### 2.4.1. Hippocampus homogenate preparation

After the behavioral tests, rats have sacrificed under ketamine (150 mg/kg) anesthesia. The whole brain was isolated, and the hippocampus was excluded from it, cleaned of excess tissues, washed with ice-cold phosphate-buffered saline (PBS) (pH 7.4), and hippocampus homogenized in cold Tris–HCl buffer solution (150 mM, pH 7.4 and protease inhibitor). After centrifuging (1,000 *g*, 4°C, 10 min), the supernatant was used for the following assays. All parameters were measured in duplicate. The Bradford method was applied to determine the total protein (Bradford, 1976).

#### 2.4.2. Measurement of hippocampus SOD activity and GSH content

Superoxide dismutase (SOD) activity was determined using a specific assay kit (Kiazist, Live Science, Tehran, Iran). In brief, the supernatant was incubated with xanthine and xanthine oxidase in potassium phosphate buffer for 30 min, and nitroblue tetrazolium (NBT) was added. Blue formazan formation was monitored at 550 nm (*n* = 5/group). Reduced glutathione (GSH), a non-enzymatic intracellular defensive element, was determined using a specific assay kit (Kiazist, Life Science, Iran). Briefly, the supernatant was mixed with 5% trichloroacetic acid and centrifuge, then 0.1 ml of obtained supernatant, 2 ml of phosphate buffer (pH 8.4), 0.5 ml of 5′5 dithiobis (2-nitrobenzoic acid) (DTNB), and 0.4 ml of distilled water were added. After 30 min incubation, the absorbance was read at 412 nm (Ellman, 1959).

### 2.5. Mitochondrial estimation

#### 2.5.1. Mitochondrial isolation

Mitochondria were extracted as previously described (Nazari et al., 2022). Briefly, rat brains were rapidly removed and minced in an isolation buffer (70 M sucrose, 230 mM mannitol, 1.0 mM EDTA, and 10 mM Tris–HCl, pH 7.4). After homogenizing in an isolation buffer, the suspension was centrifuged (700 × *g* for 10 min at 4°C). The supernatant fraction was collected and centrifuged again at 8,000 × *g* for 10 min. Extracted mitochondria were stored in an isolation buffer. The Bradford method assayed mitochondrial protein content (Bradford, 1976). To consider the effect of Na^+^ ions on brain mitochondrial activity, we replaced 230 mM mannitol with 165 mM mannitol and 35 mM NaCl.

#### 2.5.2. Assay of brain mitochondrial ROS

Brain mitochondrial ROS detected using a Multi-Mode Microplate reader (synergy HTX) using 2′, 7′-dichlorofluorescein diacetate (Pipatpiboon et al., 2012). Isolated mitochondrial samples (0.8 mg/ml protein) were added to microplate wells and then incubated with 2 μM 2′, 7′-dichlorofluorescein diacetate for 20 min. The fluorescence was measured at 490 nm excitation and 530 nm emission wavelength.

#### 2.5.3. Mitochondrial membrane potential detection

Mitochondrial membrane potential (Δψ_*m*_) was measured by quantitating rhodamine 123 (Rh 123) quenching. In any group, the mitochondrial fractions were incubated with Rh 123. After 5 min, Rh 123 (a cationic fluorescent dye) was excited at 490 nm and detected at the emission wavelength of 530 nm (Luo and Shi, 2005). A fluorescent microplate reader measured the change in Δψm as fluorescence intensity.

### 2.6. Electrophysiological recordings and PP induction

Animals were anesthetized with urethane (1.5 g/kg, i.p.) and placed in a stereotaxic frame fitted with ear cuffs to restrain head movement. Head skin and connective tissue on the skull were removed to make the sutures visible. A stainless steel bipolar stimulating electrode was placed in the perforant path [–6.9 mm posterior and ± 4.1 mm lateral to the bregma, –1.7 mm (from dura) ventrally], and the evoked potentials were recorded extracellularly with a stainless-steel electrode from the cell body layer of the dentate gyrus (DG) [–3.1 mm posterior and ± 2.1 mm lateral to the bregma, –2.7 mm (from dura) ventrally 1.5–1.8]. The responses were amplified 1,000×, digitized at 10 kHz, and filtered at a band of 0.1 Hz–10 kHz (Science Beam Co., Iran). Applied stimuli were biphasic square waves with a width of 200 ms. Signals were passed through the Analog-Digital interface to a computer, and data were analyzed using software (Eprobe Science Beam). Before LTP induction, the stimulus-response curves are executed using a range of stimulus intensities (100–1,200 μA), and stimulation intensity was adjusted at a level to evoke 40% of the maximal response [Population Spike (PS) and field Excitatory Post-Synaptic Potential (fEPSP)]. Then stable stimulus-response curve baseline at varying current intensities was obtained for at least 30 min. LTP was induced through high-frequency stimulation (HFS) by 10 trains of 10 pulses at 200 Hz separated by 10 s. After the tetanic stimuli, the PSs were recorded for the next 60. Five consecutive evoked responses were averaged at stimulus intervals of 10 s. The mean PS amplitudes and Slop during the period (0–30 min) were normalized to 100%, and the relative PS amplitudes or slop at every point were normalized relative to the sham period.

After recording a 30–40 min baseline, the paired-pulse depression/facilitation was determined. The response to the paired-pulse stimulation was subsequently recorded and delivered at 40% maximal stimulus intensity with Inter-Pulse Intervals of 20, 30, 50, 70, 100, and 120 ms. For each Inter-Pulse Interval, ten consecutive evoked responses were averaged. The fEPSP slope ratio (percentage of second fEPSP slope/first fEPSP slope; fEPSP2/fEPSP1%) and the population spike amplitude ratio (PS2/PS1%) were determined at various inter-stimulus intervals.

### 2.7. Histology tests

#### 2.7.1. Congo red and nissl staining

Histological studies were done on the hippocampal samples of randomly chosen rats in each group (*n* = 5 for each staining). After LTP recording, the rats were sacrificed by decapitation under urethane anesthesia and transcardially perfused with 80 ml of normal heparinized saline and 70–80 ml of a fixative solution containing 4% paraformaldehyde in 0.1 M phosphate buffer (PBS, pH = 7.4). After perfusion, the animals were decapitated, and brains were removed from the cranium and kept in 10% formaldehyde solution at ambient temperature for 24 h. Then hippocampus portion of the brain containing the CA1, CA2, CA3, and DG was removed according to the following coordinates: –1.5 anterior to –6.5 posterior to Bregma (Paxinos and Watson, 2006) and processed for light microscopic study. For tissue processing, the samples were dehydrated with an ascending ethanol series, cleared with xylene, and embedded in paraffin. Finally, the hippocampal block was cut into 10 μm coronal sections and prepared for Congo red (To count and identify β-amyloid plaques) and Nissl (for stereology and measurement of neuronal cell density) staining. Amyloid-β plaques were measured in the hippocampal areas, including CA1, CA2, CA3, and DG.

#### 2.7.2. Stereology and measurement of neuronal cell density

After Nissl (Cresyl violet acetate) staining, a physical disector was used to estimate the cell number of neurons. Counting cells should be considered that the neurons were distinguished from the glial and non-neuron cells by their larger cell bodies with visible euchromatin nuclei and prominent nucleolus (Ling et al., 1973). Ten sections were chosen from each rat by systematic random sampling, and four scattered representative fields were selected in the four directions of each section at an equal distance in the x–y plane. The numerical density of cells was estimated with Nv = [ΣQ-/(h × a/f × Σp)]. ΣQ-is the number of the whole cells counted in all the fields, h is the height of the optical disector, a/f is the area of the counting frame, and Σp is the total number of the counted frames in all fields. Cell counting was performed by an observer who was blinded to the condition.

### 2.8. Data analysis

All experimental analysis was performed blind. Statistical analysis was undertaken using Prism 8 for Windows (Graph Pad Software, USA). The Kolmogorov–Smirnov test was administered to determine the normal distribution of data. The one-way ANOVA or two-way ANOVA with Tukey’s *post-hoc* test fulfilled the intergroup comparison. Results have been shown as Means ± Standard Error of mean (S.E.M(. All statistical comparisons considered differences statistically significant at a *P*-value below 0.05.

## 3. Results

### 3.1. Neurobehavioral observations

#### 3.1.1. Effect of α-Tocopherol on cognitive function of rats with ICV-STZ in Morris water maze task

**FIGURE 2:**
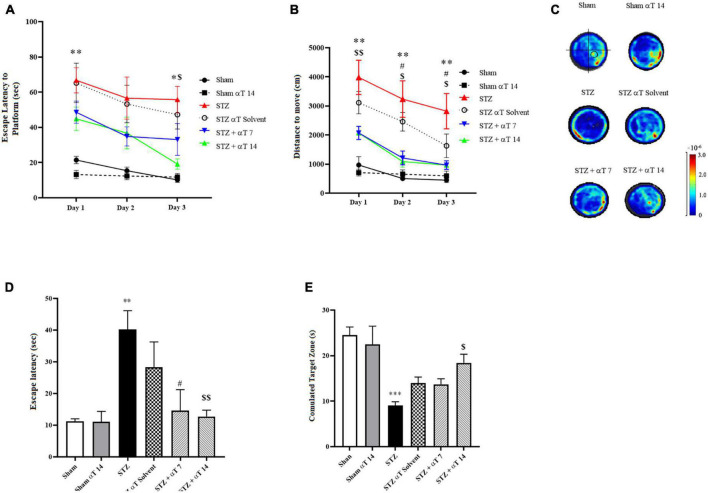
Effect of α-Tocopherol on learning and memory in icv-STZ induced rats by using Morris water maze (MWM) test. A Two-way ANOVA of **(A)** escape latency (the time to find the hidden platform) (*P < 0.05, **P < 0.01 icv-STZ compared to the sham group; ^$^*P* < 0.05 icv-STZ compared to the STZ + α-Tocopherol 14) and **(B)** distance traveled indicated that the performance of icv-STZ induced rats was inferior to that of sham and α-Tocopherol rats during place training (**P < 0.01 icv-STZ compared to the sham group; ^#^*P* < 0.05 icv-STZ compared to the STZ + α-Tocopherol 7 and ^$^*P* < 0.05, ^$$^*P* < 0.01 icv-STZ compared to the STZ + α-Tocopherol 14). **(C)** Heat maps of the swim patterns during probe testing, as examples. A black circle in the bottom right quadrant of the heat map indicates where the platform is located. **(D)** During the probe trial, icv-STZ-induced rats spent more time finding the hidden platform at the former location of the platform than did the sham and groups [icv-STZ compared to sham (**P < 0.01), STZ + α-Tocopherol 7 (^#^*P* < 0.05) and STZ + α-Tocopherol 14 (^$$^*P* < 0.01) groups]. **(E)** During the probe trial, icv-STZ rats spent less time in the target quadrant searching for the missing platform than the sham (***P < 0.001) and STZ + α-Tocopherol 14 (^#^*P* < 0.05) groups. The pattern of rat movement in MWM. Data are presented as mean ± SEM (*n* = 9 rats in each group).

We explored spatial learning ability, the spatial memory ability induced by icv-STZ, and the consumption of α-Tocopherol in rats by the MWM test. Escape latency to find the hidden platform and travel distance during the acquisition phase in MWM is presented in Figure 2. Repeated measures two-way ANOVA revealed that the performance of rats in all groups progressively improved during the days 1–3 training period and time trend analysis indicated significant differences between study groups [*F*_(1_._493_,_11_._95)_ = 17.79; *P* < 0.001 (*n* = 9) in Escape latency to find the hidden platform and *F*_(1_._412_, _11_._30)_ = 11.95; *P* < 0.01 (*n* = 9) in travel distance]. Results of latency to reach the platform during the learning phase revealed that in comparison to the STZ injection group, the escape latency was markedly shorter in the sham group and α-Tocopherol receiving groups, particularly during 1st and 3rd days that exhibited shorter latency. These differences were statistically significant (*P* < 0.05; Figure 2A). The repeated-measures ANOVA indicated that the rats in groups of control, sham + α-Tocopherol 14, STZ + α-Tocopherol 7, and STZ + α-Tocopherol 14 progressively improved their ability to locate the platform over the 3 days of training, indicating that they learned the spatial navigation task.

Meanwhile, rats in STZ and STZ + solvent groups had a significantly longer distance to find the platform than other groups on the 1st to 3rd day, indicating the harmful effects of STZ on the learning process (Figure 2B). The results of the probe-trial tests are presented in Figures 2D, E. A probe trial test, was conducted to assess memory retrieval 24 h after the last training. Two parameters of latency to the first reach to the platform location and the total time spent in the target zone were analyzed. The one-way ANOVA applied to the data obtained from the probe test revealed that STZ had a significant effect on latency in the first entry to the platform location [*F*_(5,42)_: 3.448; *P* < 0.001; *n* = 8] and the total time spent in the target quadrant [*F*_(5,36)_: 6.696; *P* < 0.001; *n* = 8]. The statistical assessment confirmed that the STZ group had a higher latency upon first entry to the platform location compared to the sham group (*P* < 0.01) (Figure 2D). Compared between the STZ group and α-Tocopherol groups (STZ + α-Tocopherol 7 and STZ + α-Tocopherol 14) indicated that there was a significant difference between the STZ groups compared to the α-Tocopherol groups for the latency upon first entry to the platform location (*P* < 0.05 in STZ + α-Tocopherol 7 and *P* < 0.01 in STZ + α-Tocopherol 14). Our results also showed a significant difference between the STZ and the sham groups for the total time spent in the target quadrant (*P* < 0.001). Statistical analyses indicated less time was spent by the STZ + αT 14 groups compared to the STZ group (*P* < 0.05). Comparing the heat map between the groups also confirms these results (Figure 2C).

#### 3.1.2. Effect of α-Tocopherol on cognitive function of rats with ICV-STZ in novel object recognition task

**FIGURE 3:**
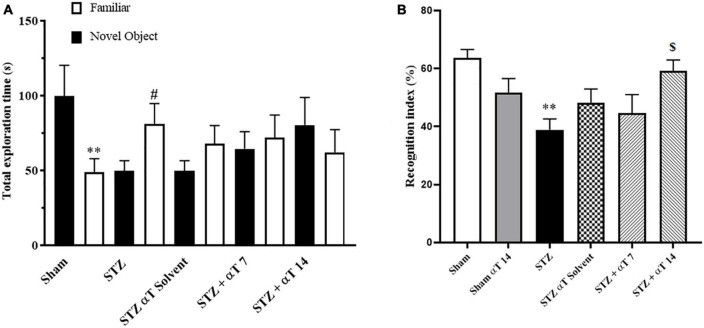
Performance on the NOR task for total exploration time and recognition index. **(A)** One-way ANOVA indicated a significant difference between the icv-STZ groups and the sham group for the whole exploration time (**P < 0.01 in the sham and ^#^*P* < 0.05 in the icv-STZ group; there is no significant difference in the treatment groups). **(B)** One-way ANOVA indicated a significant difference between the icv-STZ groups and the sham group on the recognition index (*P* < 0.05). Tukey’s *post-hoc* analyses indicated sham, and α-Tocopherol (STZ + α-Tocopherol 14) groups animals spent less time on familiar objects compared to novel objects (**P < 0.05 between sham versus icv-STZ and ^$^*P* < 0.05 STZ versus STZ + α-Tocopherol 14 groups) (*n* = 9). All graphs were plotted as mean ± SEM.

The NOR test’s preference to explore the novel object reflects the animal’s learning and recognition memory. This study measured performance on the NOR task by the total exploration time and recognition index. The one-way ANOVA applied to the data obtained from the NOR test revealed a significant effect on the recognition index [*F*_(5,51)_: 4.741; *P* < 0.01] but not total exploration [*F*_(9,80)_: 1.433; *P* = 0.188]. Our results indicated that sham group animals and STZ + αT 14 groups tended to spend more time exploring a novel object than a familiar object. This tendency was significant in the sham group (*P* < 0.01); on the contrary, in other groups, there was a tendency to spend more time exploring a familiar object than a novel object (*n* = 9; Figure 3A), especially in the icv-STZ injection group, this tendency was significant (*P* < 0.05). Our results indicated the recognition index was significantly lower in the icv-STZ injection groups (63.62 ± 2.9%) than in the sham group (38.91 ± 3.8%; *P* < 0.01) and the STZ + α-Tocopherol 14 group (59 ± 3.1%; *P* < 0.05) (Figure 3B). Our results indicated that icv-STZ injection leads to persistent impairment in recognition memory in adult rats, and treatment with α-Tocopherol repaired this persistence (*n* = 9).

### 3.2. Biochemical experiments

#### 3.2.1. Effect of α-Tocopherol on icv-STZ-induced SOD levels and GSH content in hippocampus tissue

**FIGURE 4:**
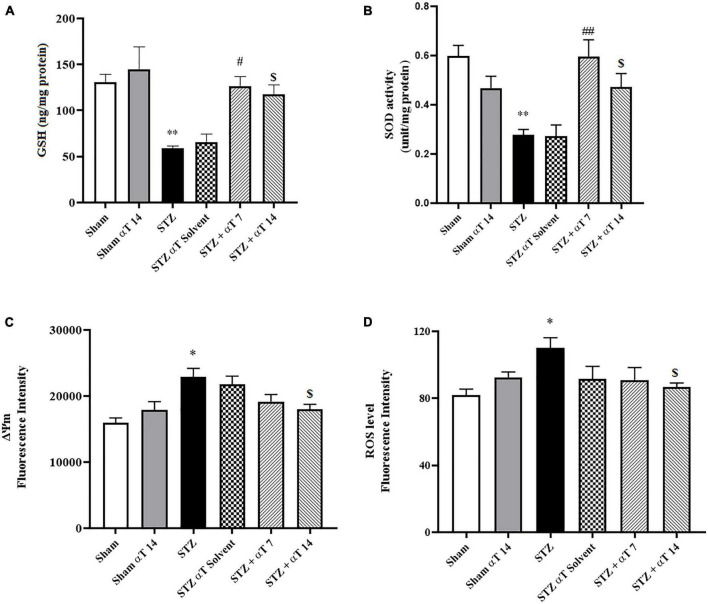
Effect of α-Tocopherol therapy on stress oxidative and mitochondrial function. **(A)** The glutathione (GSH) content and **(B)** (SOD) the superoxide dismutase activity in the hippocampus homogenate significantly increased in the icv-STZ group in comparison with those of the sham and sham + α-Tocopherol group (**P < 0.01 in sham versus icv-STZ group) treatment with α-Tocopherol (STZ + α-Tocopherol 7 and STZ + α-Tocopherol 14) increased the GSH content by comparison with an icv-STZ group (^#^*P* < 0.05 and ^$^*P* < 0.01, respectively). Treatment with α-Tocopherol also increased the SOD activity by comparison with the icv-STZ group (^##^*P* < 0.01 and ^$^*P* < 0.05, respectively), (*n* = 5). **(C)** Increased Rh 123 fluorescence intensity in the icv-STZ group (*P < 0.05 compared to sham) decreased following α-Tocopherol post-treatment (STZ + α-Tocopherol 14) [^$^*P* < 0.05 compared to the icv-STZ group (*n* = 5)]. **(D)** Increased ROS production in the icv-STZ group (*P < 0.05 compared to sham) decreased following α-Tocopherol post-treatment (STZ + α-Tocopherol 14) (^$^*P* < 0.05 compared to the icv-STZ group). All graphs were plotted as mean ± SEM (*n* = 6).

By estimating GSH contents and SOD activity, we evaluated the defense potential of the cell against oxidative stress. The one-way ANOVA applied to the data obtained from the oxidative stress tests revealed a significant effect on GSH content and SOD activity [*F*_(5,24)_: 8.19; *P* < 0.0001 for GSH and *F*_(5,24)_: 5.88; *P* < 0.0001 for SOD]. According to Figures 4A, B, in the hippocampus, the content of GSH decreased significantly in the icv-STZ group compared to the sham group (*P* < 0.01). Our results indicated that GSH in α-Tocopherol groups (STZ + α-Tocopherol 7 and STZ + α-Tocopherol 14 groups) significantly increased as compared to the icv-STZ group (*P* < 0.05 in both STZ + α-Tocopherol 7 and STZ + α-Tocopherol 14 groups; Figure 4A). Our result also indicated in the hippocampus, the activity of SOD decreased significantly in the icv-STZ group as compared to a sham group (*P* < 0.01) and in STZ + α-Tocopherol 7 and STZ + α-Tocopherol 14 groups [*P* < 0.01 in STZ + α-Tocopherol 7 and *P* < 0.05 STZ + α-Tocopherol 14 groups; (*n* = 5) Figure 4B].

#### 3.2.2. Effect of α-Tocopherol on STZ-induced mitochondrial damage

Mitochondrial damage was determined using cationic fluorophore dye rhodamine 123. Determining mitochondrial membrane potential using Rh 123 provided interesting results about the effectiveness of α-Tocopherol treatment on ΔΨm alteration. Our results indicated a significant increase in mitochondrial damage compared to the sham group *via* bilaterally injected in the hippocampus of STZ [*F*_(5,24)_: 5.60; *P* < 0.001]. As shown in Figure 4C, treatment of α-Tocopherol decreased fluorescence intensity, resembling mitochondrial protection. α-Tocopherol administration for 14 consecutive days significantly reduced mitochondrial damage compared to the icv-STZ group (*P* < 0.05). We also observed no significant effect in pre-treatment (STZ + α-Tocopherol 7) compared to the icv-STZ group (*n* = 5 per group) (Figure 4C).

#### 3.2.3. Effect of α-Tocopherol on STZ-induced mitochondrial ROS generation

We examined the level of ROS to confirm the mitochondrial dysfunction induced by the icv-STZ injection. In the next step, we investigated whether α-Tocopherol was solely against ROS produced from the isolated mitochondria of the hippocampus tissue. The one-way ANOVA applied to the data obtained from the mitochondrial ROS generation revealed a significant effect on mitochondria ROS [*F*_(5,30)_: 8.19; *P* < 0.05]. As shown in Figure 4D, the mitochondrial generation rate of ROS was significantly enhanced in animals of the icv-STZ group relative to the sham group (*P* < 0.05). Our results indicate that administration of α-Tocopherol for 14 consecutive days attenuated the increment of ROS production induced by STZ in rats (*P* < 0.05), but the reduction of ROS levels in the STZ + α-Tocopherol 7 group was not significant compared to icv-STZ group (*n* = 6).

### 3.3. Electrophysiological experiments

#### 3.3.1. Effect of α-Tocopherol on long-term potential

**FIGURE 5:**
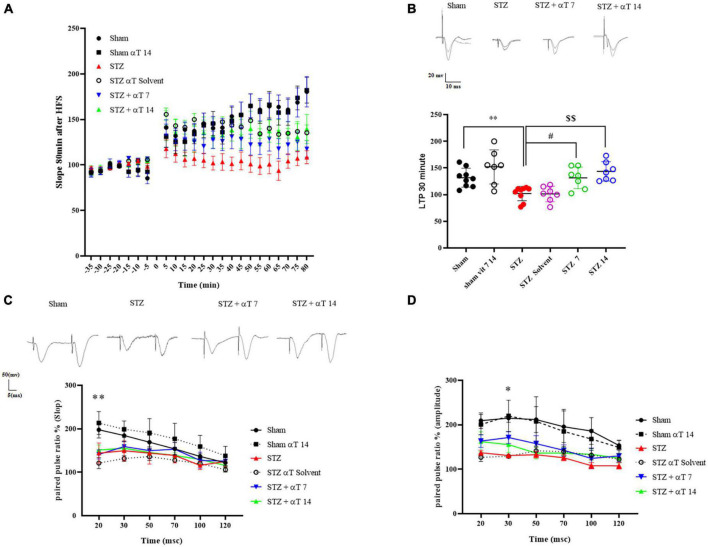
Effect of α-Tocopherol therapy on neuronal synaptic plasticity in icv-STZ induced rats. **(A)** The normalized EPSP slope in different groups before and after HFS. In this respect, the EPSP slope was lower in icv-STZ animals, and they recovered in the αT-treated group. **(B)** Specimen recordings show changes in LTP recording for 60 min after HFS (**P < 0.01 STZ in comparison with sham; ^#^*P* < 0.05 and ^$$^*P* < 0.001 STZ + α-Tocopherol 7 and STZ + α-Tocopherol 14 in comparison with STZ, respectively). The upper panel shows recorded traces from different groups. **(C,D)** The effect of icv-STZ on paired-pulse responses in the hippocampal dentate gyrus. At the intervals of 20, 30, 50, 70, 100, and 120, as shown by the field Excitatory Post-Synaptic Potential (fEPSP) slope ratio and Population Spike (PS) amplitude ratio (second response/first response ratio) (*n* = 6 per group). There was a significant difference in the paired-pulse ratio of PS amplitude and slop in an icv-STZ group compared to the sham group, especially in intervals of 20 and 30 msec, respectively (*P < 0.05, **P < 0.01 STZ in comparison with sham). Traces were recorded at the dentate gyrus at an inter-stimulus interval of 30 ms.

Figure 5 shows the effect of α-Tocopherol treatment on LTP induction and maintenance in the dentate gyrus of induced icv-STZ rats. Basal synaptic transmission was studied by analyzing short- and long-term potentiation by assessing pair pulse facilitation (PPF) and LTP. High-frequency stimulation (400 Hz) of the medial perforant path produced a long-lasting synaptic potentiation in a sham group compared to the icv-STZ group up to 80 min after HFS. A repeated measure Two-way ANOVA followed by Tukey’s *post-hoc* test revealed an interaction between time and groups in fEPSP-LTP slop after tetanization (HFS) [*F*_(110_,_659)_: 3.126, *P* < 0.001]. The one-way ANOVA applied to the data obtained from fEPSP records revealed a significant effect on fEPSP-LTP slop [*F*_(5,40)_: 8.52; *P* < 0.05]. Our results indicated a significant decrease of fEPSP-LTP slop in the icv-STZ group relative to the sham and sham + α-Tocopherol 14 groups (*P* < 0.01). Moreover, in α-Tocopherol treated (STZ + αT 7 and STZ + αT 14) animals, tetanic stimulation induced a significant increase of fEPSP-LTP slop observed compared to icv-STZ group (*P* < 0.05 in STZ + α-Tocopherol 7 and *P* < 0.01 in STZ + α-Tocopherol 14 group) (Figures 5A, B). Similar results were observed for the PS amplitude by assessing LTP (data not shown).

#### 3.3.2. The effect of α-Tocopherol on paired-pulse plasticity

As shown in Figure 5, in short intervals between two strict pairs of stimuli, the amplitude of PS and slop prompted by the paired-pulse in the icv-STZ injection group did not increase compared to the sham group. Two-way ANOVA with Tukey’s *post-hoc* analyses indicated a significant difference in the paired-pulse ratio of PS amplitude and slopped in the icv-STZ group compared to the sham group, especially in intervals of 20 and 30 msec. In other intervals, our results indicated no significant difference in the PS amplitude and slopped paired-pulse ratio between icv-STZ and treatment groups (α-Tocopherol 7 and αT 14) (*n* = 6 per group).

### 3.4. Effects of α-Tocopherol on histopathological analysis

**FIGURE 6:**
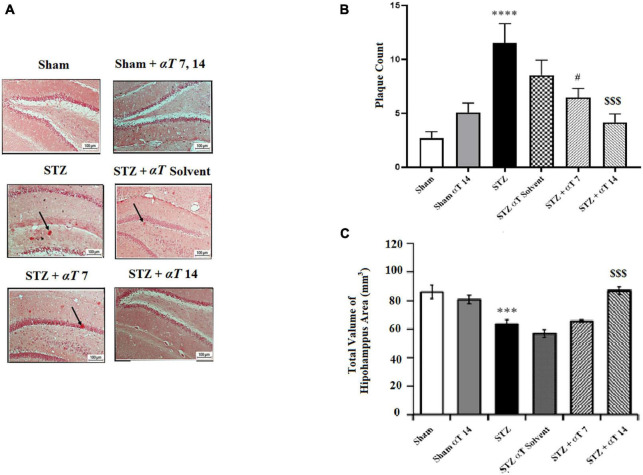
The number of β-Amyloid plaques and measurement of neuronal cell density in the hippocampal areas. **(A)** Congo red staining in the hippocampus area for sham, sham + αT 7, 14), STZ, STZ + solvent, STZ + αT, 7 and STZ + αT 14. Black arrows show β-Amyloid plaques. **(B)** Results of congo red staining for β-Amyloid plaques (****P < 0.0000 STZ in comparison with sham; ^#^*P* < 0.05 and ^$$$^*P* < 0.001 STZ + α-Tocopherol 7 and STZ + α-Tocopherol 14 in comparison with STZ, respectively). **(C)** The number of Nissl-stained neurons in hippocampus areas in experiment groups. This assessment was performed 4 weeks after icv-STZ injection (*n* = 5 in each group) (***P < 0.001, STZ in comparison with sham; ^$$$^*P* < 0.001; STZ + α-Tocopherol 14 in comparison with STZ) (*n* = 5). All graphs were plotted as mean ± SEM.

β-Amyloid plaque formation in the hippocampus is shown in Figures 6A, B. Accumulating Aβ in the hippocampus is a significant factor in the progress of sAD pathogenesis. The presented study used Congo red staining to determine the Aβ peptide accumulation. The one-way ANOVA applied to the data obtained from the Congo red revealed a significant effect on Aβ peptide accumulation [*F*_(5,56)_: 8.66; *P* < 0.0001]. Our results showed that icv-STZ injection into normal animals resulted in significant Aβ peptide accumulation. According to this, the number of plaques in the hippocampus in the STZ and STZ + solvent groups was significantly more than in sham animals (*P* < 0.0001). Our results indicated that α-Tocopherol markedly reduced Aβ plaque in the test rats compared to those in the icv-STZ group, and there is a significant difference between the STZ group compared to STZ + α-Tocopherol 7 (*P* < 0.05) and STZ + α-Tocopherol ocopherol 14 groups (*P* < 0.001) in the number of plaques. As shown in Figure 6C, the process of neuronal death in the hippocampus areas was assayed by Nissl staining. Results revealed that there is a significant decrease in the number of neurons of dentate gyros of the hippocampal regions STZ and STZ + solvent compared to the sham (*P* < 0.001) and α-Tocopherol 14 groups (*P* < 0.001). There were not any significant differences in the number of neuron cells in the α-Tocopherol 7 group compared to STZ groups (*p* > 0.05) (*n* = 5 per group).

## 4. Discussion

Icv-STZ injection (2–3 mg/kg) is a common experimental technique for sporadic AD investigations because it alters brain glucose and energy metabolism. This model results in pathological changes associated with sAD, such as the buildup of phosphorylated tau protein and beta-amyloid in the cerebral cortex tissue, dramatically increasing oxidative stress in the brain (Moreira-Silva et al., 2019; Sharma et al., 2019). Recent investigations have revealed a connection between reduced glucose metabolism *via* disease development and cognitive deficits in sAD animal models, in contrast to previous research that shows a direct association between brain glucose metabolism and function and neuron survival (Chen et al., 2021). In the current investigation, oxidative stress, disturbed homeostasis, and mitochondrial dysfunction were induced by an icv injection of STZ. Our findings showed that in brain tissue, α-Tocopherol reduces oxidative stress, neuronal loss, and Aβ plaque aggregation. In the icv-STZ-caused sAD model, α-Tocopherol can be a promising possibility for lowering glucose metabolism deficits and suppressing reactive oxygen species due to its powerful antioxidant properties and ability to decrease apoptosis. In the present study, we used icv injection of STZ to induce oxidative stress, disrupted homeostasis, and mitochondria dysfunction (Butterfield and Halliwell, 2019). These alterations also show the significant role of α-Tocopherol in improving memory and cognition. Nevertheless, more research is required to identify the exact mechanisms underlying the α-Tocopherol beneficial effects of regulating glucose metabolism in the brain and its different areas.

It is known that α-Tocopherol can cross the blood-brain barrier (BBB) and accumulate in the brain following both intra-peritoneal (Cecchini et al., 2003; Ferri et al., 2003) and oral administration (Murray and Lynch, 1998; O’Donnell and Lynch, 1998). In this respect, we used oral administration α-Tocopherol and evaluated its effects using two treatment modalities, i.e., pre-treatment and post-treatment, on the icv-STZ injection rat model. We also assayed the effects of combined injection of pre-and post-treatment α-Tocopherol. Due to the similar results with post-treatment (α-Tocopherol 14), data were not shown. In our study, mortality was ∼10% in each group of injections of STZ. Our data revealed that rats treated with 3 mg/kg STZ showed mortality of 1 rat in any group within 24 h after injection (4 rats in total).

In our study, the administration of icv-STZ significantly impaired learning and spatial memory performance in the Morris water maze. In this regard, several lines of evidence explained the damaging effects of STZ in connection to long-term object recognition memory deficits and spatial memory functions in MWM in rats 3 or 4 weeks after icv-STZ administration (Shoham et al., 2003; Rajasekar et al., 2017; Kheradmand et al., 2018; Latina et al., 2021). They corroborated the impaired retention memory elicited in icv-STZ rats during a spatial navigating task in MWM. According to the presented data, using α-Tocopherol before (pre-treatment) and after (post-treatment) injection of STZ significantly decreased the mean swim path length and escaped latency in finding the hidden platform sooner than icv-STZ animals. These results and previous reports revealed that α-Tocopherol pre- and post-treatment improved spatial learning and memory functions in MWM (Latimer et al., 2014). Novel object recognition is related to hippocampal memory (Clark et al., 2000); therefore, MWM results might predict NOR test impairment. In line with other studies, our data showed that icv-STZ injection induced a deficit in long-term (24 h after the training session) object recognition memory (Shoham et al., 2003; Kheradmand et al., 2018). The presented results revealed that the recognition index was significantly lower in the α-Tocopherol induced groups (STZ + α-Tocopherol 14) than in the icv-STZ group. Consistent with our data, Guimaraes et al. (2015) showed that α-Tocopherol could modulate memory formation and preserve cognition in the Stork model (Guimaraes et al., 2015). Sarmento-Silva et al. (2014) conducted the antioxidant effects of α-Tocopherol in improving motor and cognitive deficits induced by chronic reserpine in rats. Therefore, these data showed that α-Tocopherol could eliminate STZ-induced effects by using its antioxidant capacity and regulating glucose metabolism (as two proposed mechanisms), which is essential in improving cognition and memory function.

The present study demonstrated that icv-STZ administration induces oxidative stress, evidenced by a significant decrease in GSH and ROS levels and enzymatic activity of SOD. Damage and dysfunction of the mitochondrial membrane potential were also present. Numerous pieces of evidence suggest that oxidative stress and excessive ROS have a role in the etiology of neurodegenerative illnesses and can alter cellular components such as enzymes, proteins, and gene expression in organelles (Ray et al., 2012; Misrani et al., 2021). STZ-induced glucose and energy impairment could also be a potential source of oxidative stress (Pinho et al., 2014). ROS comprise both non-radical and radical oxygen species produced by a partial reduction of the oxygen, such as nitric oxide (NO), hydrogen peroxide (H_2_O_2_), superoxide radical anion (O_2_), etc. (Ray et al., 2012). This ROS generated during cellular respiration has detrimental effects on mitochondria, and neuronal function and increase of it causes reduction of mitochondrial ΔΨm and ATP generation through disturbance in energy metabolism and compromised dynamics and mitophagy. Finally, ROS further causes an increase in caspase activity and initiates cell death (for review Misrani et al., 2021). Also, It has been determined that the Aβ_1–42_ peptide was found to interact with many proteins in mitochondria (Pinho et al., 2014), and an increase in mitochondrial membrane permeability will lead to the release of cytochrome C into the cytoplasm and the induction of apoptosis (Takeyama et al., 2002; Machida and Osada, 2003).

Various effects of icv-STZ administration are mainly due to calcium influx. According to previous knowledge, mitochondrial impairment can result in Ca^2+^ dysregulation (Clementi et al., 1998), increasing calcium by interrupting the mitochondrial membrane potential and activating intracellular enzymes, increasing the level of cellular ROS, and eventually causing oxidative stress or obstructing mitochondrial membrane permeabilization. On the other hand, extensive research indicated that Ca^2+^ dyshomeostasis and mitochondrial dysfunction occur in neurons in the AD brain. Elevation in ROS production and loss in metabolic capabilities, including reduced ATP levels, characterize AD neurons (Misrani et al., 2021). ROS can react with organelles and biological structures and generate measurable products or dysfunction of a cell organelle, all of which can lead to neuronal death and progression of the disease (Angelova and Abramov, 2018). Our data showed that α-Tocopherol (in STZ + α-Tocopherol 14 groups) significantly enhanced the reduced SOD and GSH compared to the STZ group. We observed more significant damage to the mitochondrial membrane and an increase in ROS in the STZ group compared with the sham group. In contrast, the damage decreased in the presence of α-Tocopherol in groups (both pre-and post-treatment groups). According to these data, it may be postulated that the α-Tocopherol was effective in preventing the increase of Ca^2+^ level following icv-STZ administration and led to inhibition of ROS and oxidative stress. In addition, α-Tocopherol antioxidant effects are supported by other laboratory findings that α-Tocopherol inhibited oxidative stress in several neurodegenerative diseases, including traumatic brain injury, Alzheimer and Parkinson’s diseases (Nakashima et al., 2004; Muller, 2010; Sutachan et al., 2012; Mullan et al., 2017; Rana et al., 2020). Consistent with our data, it has been shown that administration of α-Tocopherol could reduce oxidative stress in SH-SY5Y neuroblastoma cells following amyloid-beta exposure and, so, can reduce the cytotoxicity induced by Aβ treatment (Giraldo et al., 2014). α-Tocopherol also dramatically reduces SOD and GSH levels in cells like cardiomyocytes, neurons, and lung cells following a toxic condition (Yang et al., 2010; Riccioni et al., 2012; Kryscio et al., 2013).

It is well established that neurons in the brain are highly plastic, responding to exogenous and endogenous stimuli, such as neurotransmitter fluctuations and/or behavior, emotions, etc., thus allowing an organism to learn and adapt to its surrounding environment (Wefelmeyer et al., 2016). Changes in neuronal plasticity are one of the mechanisms for neurodegeneration AD (Skaper et al., 2017). This change is directly related to greater synaptic efficacy through LTP and is used as a method in AD investigation (Fahanik-Babaei et al., 2019). To further investigate the long-term effects of α-Tocopherol and bilateral injection of icv-STZ into the hippocampus on neuronal activity, we assessed basal synaptic transmission and plasticity in the performant pathway of the hippocampal at approximately 1 month after icv-STZ was induced. In our results, electrophysiological investigations revealed a significant decrease in LTP and PPF in the hippocampus of rats of the icv-STZ group compared to sham group animals. In contrast, changes significantly in hippocampal LTP (not PPF) were found in groups of pre-and post-treatment when evaluated 23–30 days following icv-STZ infusion.

Previous studies have shown that STZ influences N-methyl-D-aspartate receptor (NMDA) receptors and their subunits, as STZ causes an increase in Ca^2+^ in the hippocampus and cortex. This increase in Ca^2+^ can cause neurotoxicity, neuroinflammation, and/or inhibition of LTP (Rai et al., 2014). According to data of the present study, including oxidative stress, mitochondrial dysfunction due to icv-STZ induced, suggests that altered mitochondrial function and apoptotic cell death are correlated to an increase in intracellular Ca^2+^ and may involve synaptic neurotoxicity. α-Tocopherol may prevent the growth of Ca^2+^ levels following STZ (icv) administration. A significant role for α-Tocopherol in the generation of LTP in area CA1 *in vitro* has been suggested by previous studies (Xie and Sastry, 1993). Yang and Wang (2008) showed that α-Tocopherol facilitated the Ca^2+^-dependent glutamate release, and the facilitation of glutamate exocytosis could be attributed to an increase in voltage-dependent Ca^2+^ influx. It is possible that STZ-induced NMDA receptor inactivation could significantly reduce activity-mediated calcium influx into neurons, which in turn would affect neuroplasticity, and this subject needs further study. Another possible mechanism of effects STZ on LTP is the inhibition of lipid peroxidation in post-synaptic neurons in the hippocampus. Changes in the membrane composition of post-synaptic, including NMDA receptors and abnormal or excess signals of NMDA receptors, may have a long-lasting negative influence over LTP induced (Molaei et al., 2020). Oxidative stress passively modulates synaptic formation and leads to impairment of synaptic plasticity, such as the induction of hippocampal LTP or facilitating paired pulls in the CA1 region. According to our data, α-Tocopherol as an antioxidant may affect the reduction of lipid peroxidation in pre- and post-synaptic neurons and cause induce LTP. This research assessed short-term plasticity in the DG by applying a paired-pulse protocol. Short-term plasticity depends on residual Ca^2+^ in pre-synaptic terminals, and STZ causes calcium imbalance in pre- and post-synaptic regions. Although, in our research, icv-STZ injection affected PS amplitude and fEPSP slope due to the application of paired-pulse stimulation protocols at IPIs of 20 and 30 ms (significantly), these difference in other IPIs was not noticeable. Other studies reported that the paired-pulse ratio (PPR) was decreased in response to STZ (Moghaddam et al., 2013; Ju et al., 2016). However, our data suggest that both the icv-STZ model and STZ + α-Tocopherol may present post-synaptic–rather than pre-synaptic–plasticity dysfunctions and may indicate brain insulin resistance does not result in pre-synaptic impairments (Srivareerat et al., 2011) which need further study.

## 5. Limitation

The use of icv-STZ and α-Tocopherol to alter mitochondrial oxidative metabolism and brain plasticity poses a constraint for the current investigation regarding the expression of the genes or proteins involved. The measurement of tissue α-Tocopherol levels at different experiment periods and the identification of α-Tocopherol in blood and cerebrospinal fluid (CSF) are two other limitations of this investigation. According to various research, α-Tocopherol gradually accumulates throughout supplementation in multiple tissues, including the liver, adipose tissue, heart, lung, skeletal muscle, and brain in numerous species, including rats, monkeys, and humans (Machlin and Gabriel, 1982; van Kempen et al., 2016; Gornicka et al., 2019). In addition, it is suggested to evaluate effects of α-Tocopherol on neuroplasticity and mitochondria alters in male and female together.

## 6. Conclusion

As a consequence of reducing mitochondrial ROS overproduction, oxidative stress, and possible membrane collapse inside the brain of rats exposed to icv-STZ, our findings showed that pre-and post-treatment with α-Tocopherol administration corrected memory impairment. α-Tocopherol’s potential role in controlling synaptic loss in neuronal injury, reducing neuronal oxidative stress, enhancing mitochondrial activities, and reducing neuronal death might be ascribed. Our research highlights the functional significance of α-Tocopherol maintenance as a potential therapeutic approach for managing neurodegenerative illnesses like AD.

## Data availability statement

The original contributions presented in this study are included in the article/supplementary material, further inquiries can be directed to the corresponding author.

## Ethics statement

This animal study was reviewed and approved by the Ethics and Research Committee of the Tehran University of Medical Sciences (IR TUMS.NI.REC.1398.056).

## Author contributions

All authors listed have made a substantial, direct, and intellectual contribution to the work, and approved it for publication.
